# Special Issue “Current Trends and Future Challenges in Assisted Reproduction”

**DOI:** 10.3390/jpm16010039

**Published:** 2026-01-07

**Authors:** Stefano Canosa

**Affiliations:** IVIRMA Global Research Alliance, Genera Livet, 10126 Turin, Italy; s.canosa88@gmail.com

## 1. Aims and Scope of the Special Issue

As the Special Issue on Current Trends and Future Challenges in Assisted Reproduction comes to a close, it is worth revisiting the rationale behind its conception. Infertility—which affects approximately 10–15% of couples worldwide and is steadily increasing in Western societies—remains a major public health concern. Over recent decades, reproductive medicine has undergone profound transformation, driven by rapid clinical innovation and technological advances rooted in translational research. Whereas the early era of assisted reproduction was largely focused on achieving pregnancy through the limited tools available, contemporary and future approaches emphasize maximizing cumulative live birth rates through individualized, patient-centered care. This shift reflects the extraordinary contributions of multidisciplinary teams who have developed new pharmacological agents, minimally invasive diagnostic and therapeutic techniques, and sophisticated laboratory technologies that have expanded the opportunities for effective and efficient infertility treatment. Against this backdrop, the aim of this Special Issue was to assemble a collection of diverse and forward-looking perspectives encompassing both clinical and laboratory dimensions of human infertility care. The contributions gathered here explore emerging advances in gamete and embryo culture and cryopreservation, novel approaches to embryo assessment—including time-lapse imaging—and the promise of strategies for implementing endometrial receptivity. The aim of this Special Issue was to provide an updated and comprehensive picture of the evolving landscape of assisted reproduction and to highlight the opportunities and challenges that will shape its future.

## 2. An Overview of Published Articles

A list of the papers that comprise this Special Issue can be found in the List of Contributions; a brief overview of each one is provided below.

The first contribution, from Maghiar L et al. (contribution 1), assessed whether differences in embryo development timing, observed via time-lapse monitoring, can help predict pregnancy success. The authors observed that while sequential development times were not statistically significant predictors, certain morphokinetic and grading parameters of embryos that successfully implanted and resulted in live birth may have the potential to predict embryo implantation and pregnancy outcomes.

The second contribution, by Guerra Cariati F et al. (contribution 2), provided an updated description of in vitro manipulation techniques, particularly sperm selection, emphasizing clinical case-specific methodology. They revealed that selecting the best spermatozoa to guarantee optimal paternal heritage means increasing the blastulation, implantation, ongoing pregnancy, and live birth rates, improving the success of IVF techniques.

The third contribution, from Bartolacci A et al. (contribution 3), assessed the association between day 3 embryo quality and clinical outcomes in blastocyst transfer policy. The authors observed no statistical differences in a β-HCG, clinical pregnancy, and live birth among the same-quality blastocysts derived from different day 3 embryo quality groups (top = A, good = B, and poor = C), suggesting that a day 3 embryo quality assessment may be unnecessary in planned freeze-all blastocyst cycles.

The fourth contribution, by Silvestris E et al. (contribution 4), investigated the few available data concerning the molecular regulation of immune checkpoint inhibitors (ICIs) therapy and their resulting gonadal toxicity, to hypothesize the most suitable fertility preservation strategy for patients receiving these drugs.

Zaha IA et al. (contribution 5) compared the administration of injectable and infusible PRP during endometrial preparation in IVF patients with thin endometrium and recurrent implantation failure. Compared to PRP infusions inside the uterus, sub-endometrial PRP injections in frozen embryo transfer methods have a greater pregnancy rate.

In their work, Sugihara A et al. (contribution 6), evaluated whether sperm DNA fragmentation (SDF) assessment could predict clinical outcomes in an IUI-D program. SDF increased after cryopreservation and after post-thaw density gradient without affecting clinical pregnancy, live birth, and miscarriage rates. These findings provide evidence to support better selection and preparation of sperm donors tailored to the donor semen’s properties in order to maximize the chances of a favorable treatment outcome.

Peluso G et al. (contribution 7) assessed the semen parameters at diagnosis of different cancer patients before cryopreservation and after thawing. All cancer subgroups had semen findings below the WHO reference values, suggesting that sperm has diverse susceptibilities to different cancers and cryodamage. Cancer and associated treatments have epigenetic effects on patients’ semen quality; thus, timely cryopreservation should be considered a useful personalized prerogative for any patient with a cancer diagnosis.

Lin PY et al. (contribution 8) aimed to identify the risk factors associated with the failure of intrauterine platelet-rich plasma (PRP) infusion for thin endometrium in women with recurrent implantation failure (RIF). Their findings suggest that an extremely thin endometrial thickness (EMT) and a history of numerous uterine surgeries can impede successful pregnancy, even when an optimal EMT is achieved following PRP infusion.

## 3. Discussion

Our Special Issue, “Current Trends and Future Challenges in Assisted Reproduction,” is grounded in the growing recognition that infertility—which affects a substantial proportion of reproductive-age individuals and couples—demands increasingly sophisticated, individualized treatment approaches. Assisted reproductive technologies (ARTs) have evolved far beyond simply achieving fertilization: modern efforts aim to maximize cumulative live birth rates, improve safety, optimize embryo selection, and preserve fertility in medically complex contexts. The papers that comprise this Special Issue are multidisciplinary, integrating advances in sperm and embryo handling, endometrial preparation, cryopreservation, fertility preservation, and the influence of emerging therapies such as immuno-oncology—all in pursuit of more personalized, effective reproductive care.

## 4. Recent Developments

Recent research in assisted reproductive technologies (ARTs) has continued to refine our understanding of key biological and technological factors that influence success, with a strong emphasis on improving outcomes through better diagnostics and interventions. One area of sustained interest is the assessment of sperm quality beyond conventional semen analysis. Sperm DNA fragmentation (SDF) has emerged as an important biomarker: systematic reviews and recent clinical studies suggest that elevated levels of SDF are associated with reduced fertilization rates, poorer embryo development, and diminished implantation and live birth outcomes in ART, although the predictive value for clinical pregnancy remains subject to debate due to methodological variation across studies and lack of consensus on optimal testing thresholds [1].

Parallel to SDF research, sperm selection techniques beyond basic swim-up and density-gradient centrifugation have been explored to improve the selection of spermatozoa with better functional and genetic profiles. Reviews highlight the transition to more advanced methods such as Magnetic-Activated Cell Sorting (MACS) and Intracytoplasmic Morphologically Selected Sperm Injection (IMSI), which aim to approximate physiological selection mechanisms and reduce the use of sperm with apoptotic markers or suboptimal morphology. However, despite intensive research, a clear consensus on a superior technique has yet to materialize, and clinical outcomes vary widely between studies [2].

In the context of endometrial receptivity and implantation, adjuvant therapies have been studied to improve the uterine environment, particularly in women with thin endometrium or recurrent implantation failure. Systematic reviews and meta-analyses indicate that treatments such as platelet-rich plasma (PRP) and granulocyte colony-stimulating factor (G-CSF) may increase endometrial thickness and clinical pregnancy rates, although evidence on live birth outcomes remains limited and further high-quality trials are needed to clarify efficacy and safety [3].

Finally, traditional morphological grading systems evaluate blastomere symmetry, cell number, cytoplasmic appearance, and degree of fragmentation at the cleavage stage (Day 2–3), as well as inner cell mass (ICM), trophectoderm (TE) quality, and degree of expansion at the blastocyst stage. However, the role of day 3 morphology has been the subject of renewed scrutiny. Recent evidence indicates that day 3 morphology is only a weak predictor of ultimate embryo viability, especially in clinics that routinely culture embryos to the blastocyst stage, suggesting that early morphology may reflect transient developmental dynamics rather than stable biological potential [4].

Together, these developments underline a multifaceted effort to understand and optimize the biological and technological determinants of reproductive success—even as key questions remain with regard to translating experimental and computational advances into broadly applicable clinical practice.

## 5. Gap in Knowledge

Despite these advances, substantial uncertainties and limitations persist, warranting cautious interpretation and further investigation.

Although newer sperm-selection and manipulation techniques are promising, the clinical evidence remains limited. For example, while studies have revealed associations between sperm quality (including DNA integrity) and embryo development, a recent systematic review found that sperm DNA fragmentation often correlates with reduced fertilization, lower blastocyst rates, and diminished embryo quality—but translation into consistent improvements in live birth rates remains unclear [5]. This gap in evidence means that even sophisticated sperm-selection approaches may not guarantee better clinical outcomes in all contexts.

Similarly, while PRP therapy for improving endometrial receptivity appears encouraging, data are scarce and largely derived from small, sometimes non-randomized studies. The heterogeneity in patient populations, PRP preparation and administration protocols, and outcome measures makes it difficult to generalize results or recommend PRP as a standard of care. Thus, the potential long-term safety and optimal indications of PRP remain uncertain, especially across diverse patient profiles [6].

In the field of fertility preservation, especially in the context of immuno-oncology or new cancer-therapy regimens, the long-term effects on reproductive capacity, gamete/offspring health, epigenetic status, and overall reproductive lifespan remain poorly understood. The review included in this Special Issue highlights that while immunotherapy may impact fertility, there is a lack of robust data to guide fertility-preservation strategies for patients undergoing these treatments (contribution 4).

Regarding embryo assessment and selection, the finding that day 3 embryo quality may not influence blastocyst-stage outcomes in freeze-all cycles suggests that traditional evaluation metrics may need rethinking. Yet this raises further questions: Which embryo characteristics (morphokinetics? genetic markers? metabolic profile?) truly predict implantation and live birth? And how can selection criteria be standardized across clinics? The absence of widely validated, consistent biomarkers for embryo viability remains a central limitation [7].

Finally, a broader translational gap remains: many of these innovations—while biologically plausible and promising—lack large-scale, multicenter, randomized studies with live birth, perinatal, and long-term follow-up. Without such data, long-term safety, generalizability, cost-effectiveness, and real-world applicability remain uncertain.

## 6. Special Issue Results

This Special Issue presents a rich and nuanced picture of where assisted reproduction currently stands—and where it may be heading.

The study “Correlation between Human Embryo Morphokinetics Observed through Time-Lapse Incubator and Life Birth Rate” explored whether time-lapse developmental timings could distinguish embryos that implant from those that do not. Although no morphokinetic parameter reached statistical significance, embryos that resulted in live births tended to show slightly shorter developmental intervals. Importantly, conventional embryo grading—rather than timing variables—showed a highly significant association with implantation outcomes, indicating that morphology still outperforms morphokinetics as a predictive tool in this setting (contribution 1).

The review “Advanced Sperm Selection Techniques for Assisted Reproduction” offers a comprehensive update on in vitro sperm manipulation and selection, arguing that a more tailored approach to sperm preparation—adapting the technique to the specific pathology (e.g., severe oligozoospermia, cryptozoospermia, or cancer-related infertility)—may improve outcomes. The authors argue that beyond conventional semen analysis, enhanced selection criteria and advanced preparation methods may better ensure “optimal paternal inheritance”, potentially raising rates of blastulation, implantation, and live births (contribution 2).

From the embryo-culture perspective, the retrospective study assessing the relevance of day 3 embryo quality in planned freeze-all blastocyst cycles offers provocative evidence. Among 1074 frozen–thawed single-blastocyst transfers from first ICSI freeze-all cycles, no significant differences in β-hCG positivity, clinical pregnancy rate, or live birth rate were found across blastocysts derived from day 3 embryos previously rated as top-quality, good-quality, or poor-quality. This suggests that for blastocyst-stage transfers in cryopreserved cycles, early embryo morphology (day 3) may not provide meaningful predictive value. This finding has potential implications for embryo selection protocols (contribution 3).

Moreover, the broader context of modern cancer treatments—including immuno-oncology—raises new challenges for reproductive medicine. The review “Fertility Preservation in the Era of Immuno-Oncology: Lights and Shadows” draws attention to possible gonadal toxicity, hypothalamic–pituitary–gonadal disruptions, and the lack of robust data on long-term reproductive outcomes in this setting (contribution 4).

On the endometrial side, the study “Autologous Platelet-Rich Plasma (PRP) in Infertility—Infusion versus Injectable PRP” directly compares two modalities of PRP administration in women undergoing frozen embryo transfer with previously thin endometrium or recurrent implantation failure. The authors report that sub-endometrial PRP injections were associated with greater increases in endometrial thickness and higher pregnancy rates than intrauterine PRP infusions, suggesting that the mode of PRP delivery may significantly influence efficacy (contribution 5).

The authors of the paper “Sperm DNA Fragmentation after Cryopreservation and Sperm Selection Has No Implications for Clinical Pregnancies and Live Births after Intrauterine Insemination with Donor Sperm” found that cryopreservation and processing procedures significantly increased SDF levels; however, these changes did not affect clinical pregnancy, live birth, or miscarriage rates across more than 400 IUI-D cycles. Female age emerged as the primary determinant of outcome. These results suggest that, within the donor setting, elevated SDF after laboratory handling does not compromise reproductive success and that current donor selection and sperm-preparation protocols remain robust (contribution 6).

In the realm of fertility preservation, “Semen Cryopreservation to Expand Male Fertility in Cancer Patients: Intracase Evaluation of Semen Quality” documents pre-treatment and post-thaw semen parameters in a cohort of cancer patients. The study confirms that while cryopreservation is a viable strategy, cryo-induced reductions in sperm viability and motility are common—particularly in testicular cancer—underlining the importance of prompt sperm banking before gonadotoxic therapy (contribution 7).

Finally, the study entitled “Factors Affecting the Potential Efficacy of Intrauterine Platelet-Rich Plasma Infusion on Thin Endometrium in Women with Recurrent Implantation Failure” showed that PRP-lysate treatment effectively increased endometrial thickness in most patients and allowed many to reach the clinically recommended threshold for transfer. Nonetheless, extremely low baseline thickness and a higher number of previous uterine surgeries significantly reduced the likelihood of successful treatment and pregnancy. These findings indicate that PRP-based approaches are promising but remain constrained by underlying uterine conditions (contribution 8).

Collectively, these results illustrate both the potential and the complexity of modern ART. On one hand, personalized sperm-selection strategies, regenerative therapies like PRP, and fertility preservation protocols offer new hope for patient-tailored treatments. On the other hand, the variability in outcomes and the need for careful evaluation—especially with regard to long-term effectiveness and safety—highlight that these innovations are not yet universally applicable and their efficacy has yet to be conclusively proven.

## 7. Future Work

Given the gaps and variability identified, future efforts in ARTs should prioritize well-designed, large-scale, multicenter, prospective randomized studies. Such studies would help validate the effectiveness, safety, and reproducibility of promising interventions, e.g., PRP-based endometrial preparation, advanced sperm selection, and fertility-preservation protocols in oncological contexts. There is also a pressing need to establish standardized protocols across clinics: for sperm preparation, embryo culture and assessment, PRP preparation and delivery, cryopreservation procedures, and fertility-preservation timing.

Additionally, long-term follow-up—not only in terms of live birth rates but also child health, epigenetic stability, and reproductive lifespan—is essential to determine the safety and broader implications of newer ART strategies. Particular attention should be paid to patient populations with special conditions (e.g., cancer survivors, patients undergoing immuno-oncology) and how novel therapies may impact reproductive potential.

Finally, researchers should continue exploring new biomarkers or predictive tools for embryo viability beyond classical morphology—for example, using morphokinetic data, metabolic profiling, genetic/epigenetic markers, or artificial intelligence approaches—to refine embryo selection and optimize success.

## 8. Conclusions

The Special Issue “Current Trends and Future Challenges in Assisted Reproduction” underscores that ART is rapidly evolving—moving from a once-standardized, “one-size-fits-all” paradigm toward more personalized, biologically informed, and context-sensitive strategies. The included studies demonstrate that advances in sperm selection, endometrial preparation, fertility preservation, and embryo assessment hold real promise to improve outcomes for a diverse range of patients.

However, the studies presented also reveal significant limitations, including insufficient high-quality evidence, variable protocols, limited sample sizes, and a lack of long-term data. Until more rigorous, standardized, and reproducible evidence is available, such innovations should be considered cautiously, and personalized interventions should be carefully tailored to individual patient conditions.

Ultimately, the path forward lies in combining biological insight, technological innovation, and robust clinical research, with the aim of realizing the full potential of personalized assisted reproduction in a safe, effective, and equitable manner.
